# Short-term mechanical support and pharmacotherapy, a new strategy in cardiogenic shock?

**DOI:** 10.1007/s12471-014-0531-2

**Published:** 2014-02-19

**Authors:** C. C. S. Tseng, S. A. J. Chamuleau, N. De Jonge, F. Z. Ramjankhan

**Affiliations:** 1 Department of Cardiology, UMC Utrecht, Heidelberglaan 100, 3584 CX Utrecht, the Netherlands; 2 Department of Cardio-Thoracic Surgery, UMC Utrecht, Heidelberglaan 100, 3584 CX Utrecht, the Netherlands

*Editorial comment on ‘* **Preventing LVAD implantation by early short-term mechanical support and prolonged inodilator therapy:** a case series with acute refractory cardiogenic shock treated with veno-arterial ECMO and optimised medical strategy’

J.J. Brugts, O. Manintveld, A. Constantinescu, D. Donker, R. van Thiel, K. Nieman, L. Jewbali, F. Zijlstra, K. Caliskan

In this issue of the Netherlands Heart Journal, Brugts et al. describe a series of cases with acute severe refractory cardiogenic shock [1]. All three patients had no relevant cardiac history and were initially referred for durable left ventricular assist device (LVAD) implantation and/or heart transplantation (HTx). Dilated cardiomyopathy was diagnosed in two patients (one with an unknown cause, one due to alcohol/toxicity), while parvo B10 viral myocarditis was confirmed by biopsy in the other patient. Veno-arterial extracorporal life support (VA-ECLS) was started because a high dose of positive inotropes and intra-aortic balloon pump (IABP) were insufficient. Low-dose beta blocker and ACE inhibition were initiated early and titrated in combination with phosphodiesterase inhibition (enoximone). In all three cases cardiac function recovered and the patients were successfully weaned from VA-ECLS without any signs of relapse in up to 3.5 years of follow-up.

The authors conclude that the combination of extracorporeal membrane oxygenation (ECMO) and early introduction of heart failure medication under the umbrella of phosphodiesterase inhibition provided momentum to survive and prevent LVAD implantation.

The introduction of extracorporal life support in the 1970s has defined the standard of care in so-called ‘crash and burn’ (INTERMACS 1) patients [2]. The clinical management of this patient category is still hampered by the high mortality rate but also by the lack of accurate guidelines to confirm the appropriate patients and timing for mechanical circulatory support [3].

The rise of durable LVADs has significantly improved survival in end-stage heart failure and has provided a successful bridge to transplantation [4]. However, results of durable LVADs as bridge to recovery are poor, with maximal sustained recovery rates of 20.5 % recorded by Birks in a large cohort of 92 patients with an aggressive supporting pharmacological regimen [5, 6]. Though infrequent, cardiac recovery is primarily seen in non-ischaemic cardiomyopathy with divided aetiology between myocarditis, peripartum cardiomyopathy and idiopathic, potentially reversible causes of heart failure [5–7].

Analysis on survival after implantation of a durable LVAD in different INTERMACS categories revealed dramatically high mortality rates in INTERMACS profiles 1 and 2, compared with less critically ill patients [8]. This finding gave rise to the current applied strategy of short-term mechanical circulatory support in ‘crash and burn’ patients [2]. Especially in potentially reversible causes of cardiogenic shock, temporary ECLS can be sufficient. When recovery does not meet explantation criteria a long-term device and/or heart transplantation is still indicated. Although the strategy of preventing LVAD implantation by early short-term mechanical support and prolonged inodilator therapy is described in the present case series as a novel approach, clinicians worldwide are already initially treating INTERMACS profile 1 patients with short-term mechanical support to prevent mortality.

To promote reversal of heart failure, different combinations of pharmacological strategies have been investigated. For example, sustained recovery was seen in the majority of a small group of patients with acute and severe non-ischaemic cardiomyopathy without active myocarditis treated with a strict pharmacological treatment consisting of the β2-agonist clenbuterol [9]. From the investigated combination of heart failure medication with enoximone, no conclusion can be drawn. Widely, the benefit of beta blockers in unstable severe heart failure is lacking evidence, due to routine exclusions in studies, and one should be extremely cautious in case of right ventricular failure [3, 10].

A recent retrospective study on early heart failure pharmacotherapy in ongoing cardiogenic shock compared patients who did and did not receive heart failure therapy within 24 h of admission. In comparison with no therapy, administration of β-blocker was significantly associated with an increased 30-day mortality and longer median duration of shock [11].

Rather than preventing the pathway of long-term LVAD support, the reported cases elegantly describe recovery of heart failure with state of the art treatment with ECLS in potentially reversible causes of cardiogenic shock. The additive value of the pharmacological regimen cannot be concluded from this series without a control group. Controlled studies are necessary to examine the value (or harm) of early introduction of heart failure medication.
